# Correction of Malpositioned Implants through Periodontal Surgery and Prosthetic Rehabilitation Using Angled Abutment

**DOI:** 10.1155/2014/702630

**Published:** 2014-05-14

**Authors:** Érica Dorigatti de Avila, Rafael Scaf de Molon, Luiz Antônio Borelli de Barros-Filho, Marcelo Ferrarezi de Andrade, Francisco de Assis Mollo, Luiz Antônio Borelli de Barros

**Affiliations:** ^1^Department of Dental Materials and Prosthodontics, School of Dentistry at Araraquara, Universidade Estadual Paulista, UNESP, Rua Humaitá 1680, 14801-903 Araraquara, SP, Brazil; ^2^Department of Diagnosis and Surgery, School of Dentistry at Araraquara, Universidade Estadual Paulista, UNESP, Rua Humaitá 1680, 14801-903 Araraquara, SP, Brazil; ^3^Department of Restorative Dentistry, School of Dentistry at Araraquara, Universidade Estadual Paulista, UNESP, Rua Humaitá 1680, 14801-903 Araraquara, SP, Brazil; ^4^Department of Social Dentistry, School of Dentistry at Araraquara, Universidade Estadual Paulista, UNESP, Rua Humaitá 1680, 14801-903 Araraquara, SP, Brazil

## Abstract

When dental implants are malpositioned in relation to the adjacent teeth and alveolar bone or in an excessive buccal or lingual position, the final prosthesis rehabilitation impairs the peri-implant health of the gingival tissues and the aesthetics of the patient. Thus, the purpose of this case was to report and discuss a multidisciplinary protocol for the treatment of a compromised maxillary tooth in a patient with an abscess in his right central incisor due to an excessive buccal implant position. The patient presented with an implant-supported provisional restoration on his right maxillary central incisor and a traumatic injury in his left central incisor. The treatment protocol consisted in (i) abutment substitution to compensate the incorrect angulation of the implant, (ii) clinical crown lengthening, (iii) atraumatic extraction of the left central incisor, and (iv) immediate implant placement. Finally, (v) a custom abutment was fabricated to obtain a harmonious gingival contour around the prosthetic crown. In conclusion, when implants are incorrectly positioned in relation to the adjacent teeth, associated with soft-tissue defects, the challenge to create a harmonious mucogingival contours may be achieved with an interdisciplinary approach and with the placement of an appropriate custom abutment.

## 1. Introduction

One of the main challenges in esthetic dentistry is the preservation and reproduction of the natural mucogingival architecture surrounding the malpositioned implants in the anterior maxilla, especially when the patient presents a high lip line [1]. There are several factors that can influence the healing around dental implants: (i) systemic conditions such as diabetes mellitus [2]; (ii) soft and hard tissue contours; (iii) implant position [3]; (iv) the manufacturer abutment designs; and (v) the design of the definitive prosthesis [4]. However, poor adaptation of the prosthesis can overload the implant system, and microgaps can be formed between the implant and abutment, which results in bacterial accumulation [5]. In some cases, the inflammation of the soft tissue is observed without bone level changes, a condition called peri-implant mucositis. However, when bone loss occurs due to the presence of bacteria in the microgap between the implant and abutment, the development of peri-implantitis will occur [6].

Preferably, implants should be installed parallel to each other and to adjacent teeth and be aligned vertically with axial forces. However, when implants are incorrectly angled or improperly positioned and soft-tissue defects exist, conventional abutments cannot be used; therefore the use of custom angled abutments is required [6]. Furthermore, some factors influence the abutment choice, especially related to metal band height and aesthetic appearance, as follows: (i) probing depth; (ii) amount of the gingival tissue; and (iii) implant platform [7, 8].

Some studies suggest that angled abutments result in increased stress on supporting implants, adjacent bone, and the prosthesis [9]. On the other hand, other studies [10] show that it is possible to correct the implant positioning using angled abutments. The authors stated that angled abutment did not adversely affect the retention of prostheses or implants and did not interfere in the survival of the rehabilitation. Tian et al. [11] evaluated whether angled abutments could decrease stress and promote better stress distribution on surrounding bone of single-unit dental implants. The authors concluded that angled abutments might result in decreased stress on adjacent bone of single-unit dental implants when implants are not placed in the ideal axial position. At the same time, from a biomechanical point of view, angled abutments may be a suitable restorative option when implants are not placed in the ideal axial position.

Thus, given the need to correct small angles and customize the connection between the implant and prosthesis, the aim of this paper was to report a case of an esthetic and functional anterior maxillary rehabilitation using periodontal surgery and custom abutments to correct the implant positioning. Moreover, the clinical implications of the use of angled abutments were discussed.

## 2. Case Report

A 57-year-old Caucasian man presented to the Department of Periodontology, School of Dentistry at Araraquara, Sao Paulo, Brazil, with an implant-supported provisional restoration on the maxillary right central incisor. The patient had no significant medical history and denied use of alcohol. He had sustained a traumatic injury involving his left maxillary central incisor, which had been treated endodontically and restored with a core and a crown. The main patient complaint was the uneven gingival level with consequent increasing of crown length in the right maxillary central incisor (Figures 1(a) and 1(b)).

Clinical examination showed an abscess in the right maxillary central incisor mainly caused by the cement retention (Figures 1(c) and 1(d)) and due to an excessive buccal implant positioning (Figures 2(a) to 2(d)). In this case, the short abutment resulted in an inappropriate marginal adaptation, favoring the accumulation of a cement line between the crown and abutment interfaces. The first treatment proposed was a local curettage allowing the abscess drainage. The standard abutment implant in the region of right maxillary central incisor was removed and replaced by an EsthetiCone abutment angled at 25 degrees with a 4 mm brace in height (Conexão Sistema de Implantes, Sao Paulo, SP, Brazil) (Figures 3(a) and 3(b)).

The periapical radiography revealed endodontic treatment in his left maxillary central and lateral incisors, root resorption of left maxillary central incisor, periapical lesion in his left lateral incisor, and the presence of a dental implant in his right maxillary central incisor with a prefabricated titanium abutment (Conexão Sistema de Protese, Sao Paulo, SP, Brazil) maintaining a fixed provisional restoration (Figure 3(c)). Also, the preoperative radiography evaluation of the left central incisor revealed a wide dentin loss in consequence of the prosthetic rehabilitation, associated with root resorption, hindering the aesthetic and functional rehabilitation of the patient. Due to uneven gingival level of the maxillary anterior teeth (Figure 4(a)), the proposed treatment for the patient was (i) removal of the provisional crown (Figure 4(b)); (ii) endodontic retreatment of left maxillary lateral incisor; (iii) prosthetic rehabilitation of the right maxillary central incisor and for the left maxillary central and lateral incisors (Figures 4(c) and 4(d)). Endodontic retreatment of left maxillary lateral incisor was performed using prefabricated glass fiber. Then, clinical crown lengthening was performed aiming to improve the aesthetics through the leveling of the gingival margins (Figures 5(a) to 5(c)).

Prior to the prosthetic rehabilitation, a longitudinal root fracture in the left central incisor was observed; thus, an atraumatic extraction of this tooth was performed under local anesthesia (Figure 5(d)) followed by curettage and root planing (Figures 6(a) and 6(b)). A 4.3 mm internal hexagon implant (Correct Master, Conexão Sistema de Protese, Sao Paulo, SP, Brazil) was immediately placed in the fresh extraction socket, with proper 3-dimensional orientation (Figures 6(c) and 6(d)) [12].

The implant was placed 3 mm below the bone crest and in a more palatine position, allowing a minimal distance of 2 mm from the buccal cortical bone (Figure 7(a)). Primary stability was achieved by the selection of implants with 15 mm length, which extended 5 mm from the apex of the extracted tooth. The final insertion torque was 45 N·cm.

A CeraOne abutment (Conexão Sistema de Protese, Sao Paulo, SP, Brazil) was installed immediately after the implant installation (Figure 7(b)). Thereafter, a new provisional crown involving right and left central incisors and left lateral incisor was prepared to peri-implant mucosa conditioning during the healing process (Figure 7(c)). After, periapical radiography was taken to verify the proper implant position in relation to the adjacent teeth and the correct adaptation of the prosthetic component (Figure 7(d)). In an attempt to personalize the abutment preparation and increase the contact between the implant and the components and considering the low cost of the component, UCLA abutments overcast in cobalt-chromium (Conexão Sistema de Protese, Sao Paulo, SP, Brazil) were selected.

After four months, the EsthetiCone and CeraOne abutments were removed in order to make a cast. From this cast, the custom UCLA abutments overcast with cobalt-chromium were prepared. One commonly used method to mitigate the poor appearance of peri-implant tissue caused by cobalt-chromium metal is to alter the metallic color using the application of porcelain on the component. Therefore, Ivoclar press ceramic was applied to obtain better aesthetic results on the pillars (Figures 8(a) to 8(d)). The copings were fabricated with IPS e.max Press (Conexão Sistema de Protese, Sao Paulo, SP, Brazil) and were injected with translucent ceramic with a lithium disilicate base (Figures 9(a) to 9(d)) in the maxillary central and lateral incisors and left canine. The injection of hot glass-ceramic generates the desired final dimensions of restoration and, after being painted with ceramic pigments, was covered with a glaze of powdered glass and subject to final firing. After 12 months, the rehabilitation shows excellent aesthetic results (Figures 10(a) and 10(b)). The three-year follow-up results demonstrate an improved clinical situation, allowing an optimal aesthetic outcome without probing depths or gingival recession (Figures 11(a) to 11(c)). Figures 12(a) and 12(b) showed the initial and final clinical situation, respectively.

## 3. Discussion

Usually, the use of parallel abutments to elaborate an implant-supported prosthesis instead of abutments is preferred. However, there are cases where it is not possible to place an implant in an ideal tridimensional position without additional surgical procedures [13–16]. Thus, the aim of this case was to report an esthetic and functional anterior maxillary rehabilitation using custom abutments to correct the implant positioning. According to Cavallaro and Greenstein [10], to restore peri-implant soft-tissue health, surrounding malpositioned implants, correct prosthetic component designs should be chosen, since the wrong choice of abutments could result in aesthetics and peri-implant commitment. Abutments can affect the implant stress and strain distribution, changing the forces transmission to the adjacent bone [10, 11].

Successful anterior esthetic of a single-crown restoration is achieved when the crown color matches the neighboring teeth, the peri-implant tissue contours simulate the gingiva around the crown, and the peri-implant tissue matches the patient natural color. In this particular case, we chose the UCLA abutment. This component has been indicated in some cases, including (i) implants positioned in the same gingival level; (ii) angled implants; (iii) presence of small interocclusal space; (iv) or if the final prosthesis cost is a preponderant factor [17]. Using UCLA custom abutments and overcasting to fabricate a duplicate titanium abutment simplifies the clinicians ability to replace a metallic abutment [17].

In this particular case, the patient presented with a deep peri-implant area and an angled implant, which contributed to the esthetics commitment. In relation to the implant position, the scientific literature is contradictory about the problems caused by angled abutments. In 2008, Kao et al. [18] observed that angled abutment up to 25 degrees can increase the stress in the peri-implant bone by 18%. This result is in agreement with Lin et al. [9], who found higher implant and cortical bone strain for a 20-degree angled abutment compared to straight abutments. On the other hand, Saab et al. [19] measured and compared the bone tension distribution around an implant in the anterior maxilla using 2 different abutments, parallel and angulated, by means of finite element analysis. The results showed that most of the tension produced on the cancellous and cortical bone was within the range that has been reported to increase bone mass and mineralization.

Another treatment option is the use of CAD/CAM technology. CAD/CAM allows the creation of duplicate abutments, whereby two precisely shaped abutments of similar or dissimilar materials can be fabricated from the same computer file. A crown that is fabricated for one of these abutments will fit the other, despite any material differences. However, this treatment alternative is still restrictive in developing countries due to the high cost to the patient [20]. Another option that offers better precision between the abutment-implant and abutment-crown is the use of UCLA overcasting. In this case, a customized abutment was modified with the application of porcelain over the coping; an injection of IPS max press prolonged the aesthetic life of the restoration and the soft tissues surrounding the implant.

Passive adaptation between the abutment and the implant and between the crown and the abutment prevents the onset of the inflammatory process that causes peri-implantitis. Interventions prior to the complete healing process in response to patient demands may result in unpredictable soft-tissue manifestations. This outcome may include an inadequate zone of attached gingiva, as well as the compression of the soft tissue surrounding the abutment from an overlying provisional prosthesis [4]. Achieving a better aesthetic outcome in the maxillary anterior area is necessary to obtain harmony between color and position of the crowns in relation to the adjacent teeth and the contour of the peri-implant tissue around the crown. Custom UCLA abutments overcast with cobalt-chromium allow for easy restoration of the gingival tissues, which offers precise adaptation, associated with an accessible cost. At the same time, the aesthetics of the metal collar can be corrected with the application of ceramic to the metal copings with IPS e.max Press and the injection of translucent, lithium disilicate-based ceramics.

In conclusion, when implants are incorrectly positioned in relation to the adjacent teeth, associated with soft-tissue defects, the challenge to create a harmonious mucogingival contours may be achieved with an interdisciplinary approach and with the placement of an appropriate custom abutment.

## Figures and Tables

**Figure 1:**
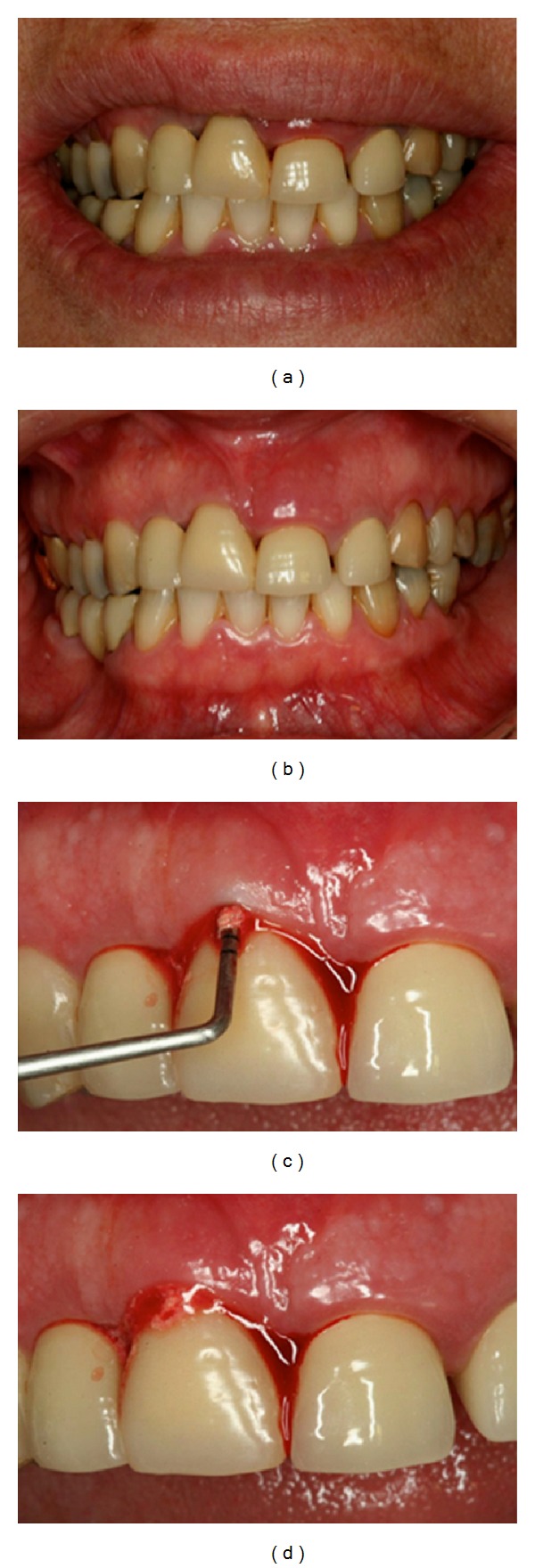
A 57-year-old female presented with an implant-supported provisional restoration on the maxillary right central incisor. (a, b) Image showed the uneven level of the gingiva of the maxillary anterior teeth. (c) Clinical examination showing abscess in the right maxillary central incisor region. (d) Cement retention of the implant-supported prosthesis.

**Figure 2:**
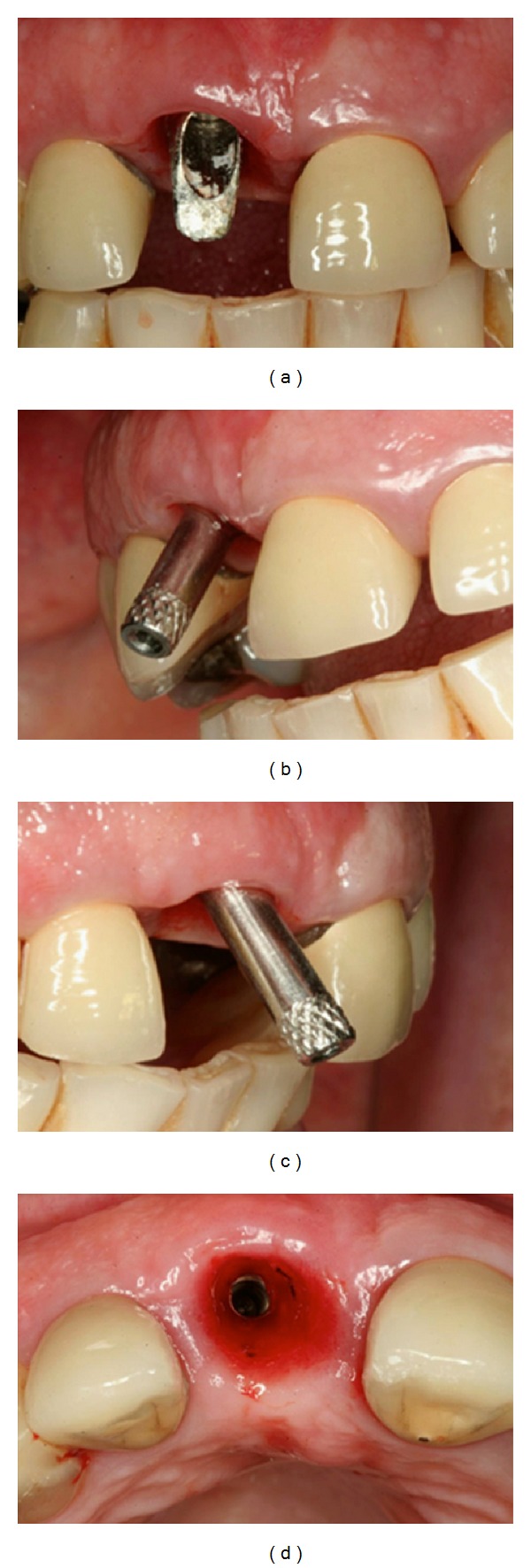
Clinical examination showing buccally implant position.

**Figure 3:**
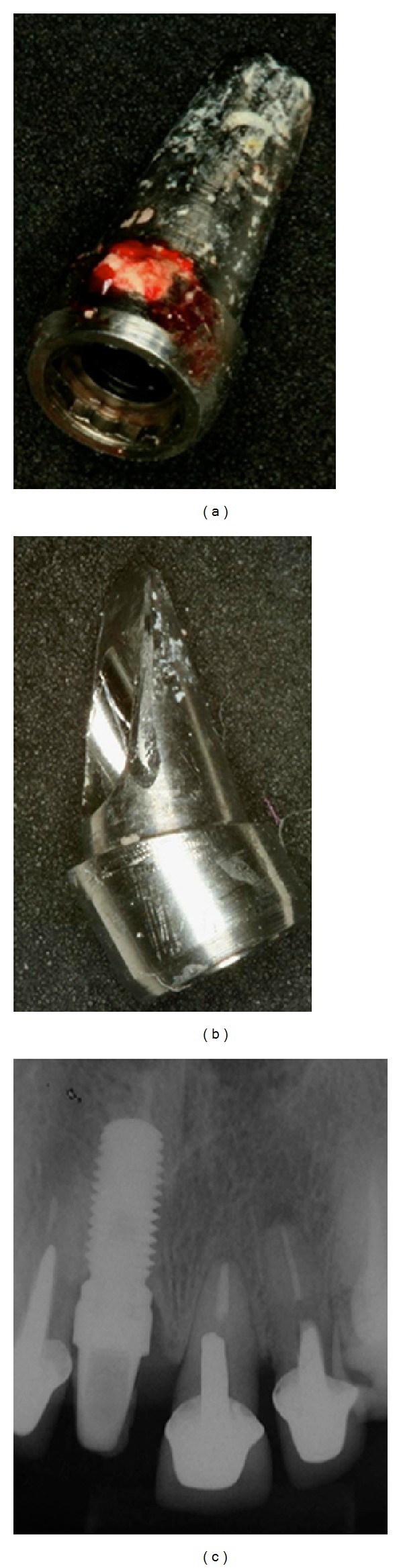
(a) Right maxillary central incisor abutment removed. (b) Angled abutment to correct the implant malpositioning; (c) periapical radiograph revealed endodontic treatment of the left maxillary central incisor and the left maxillary lateral incisor and the root resorption of left maxillary lateral incisor.

**Figure 4:**
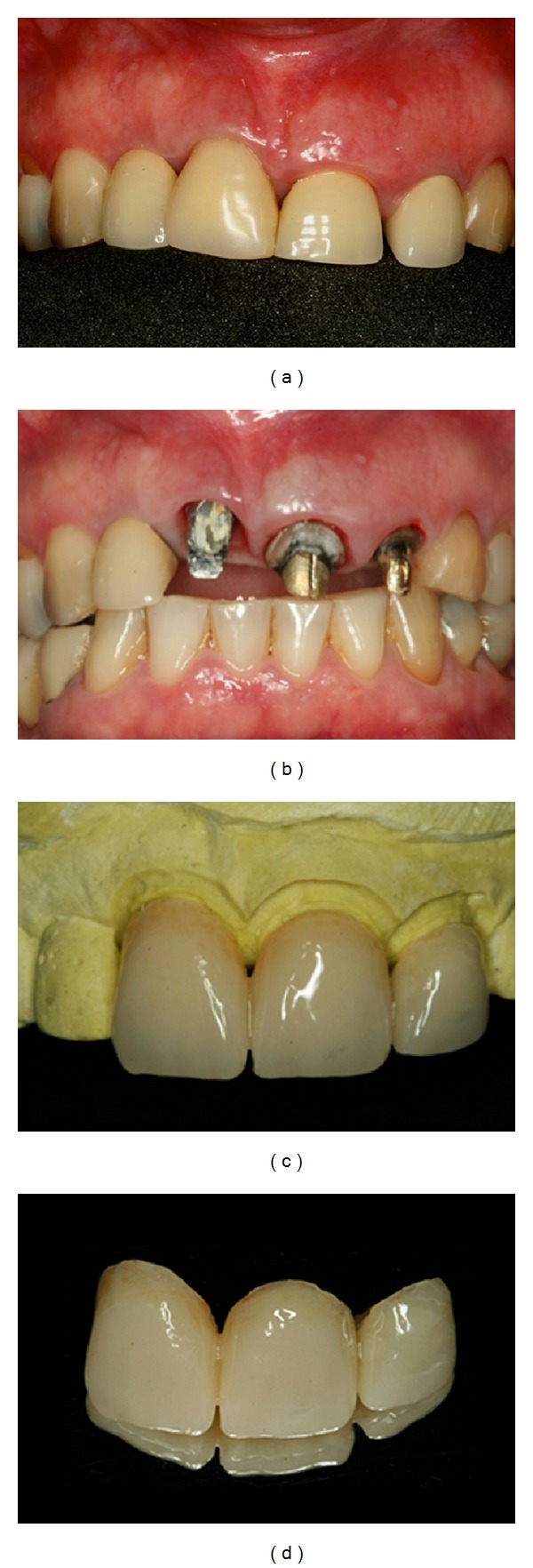
(a) Uneven gingival level of the maxillary right central incisor. (b) After removal of the provisional crown. (c, d) New provisional resin crown to favor the gingival aesthetics.

**Figure 5:**
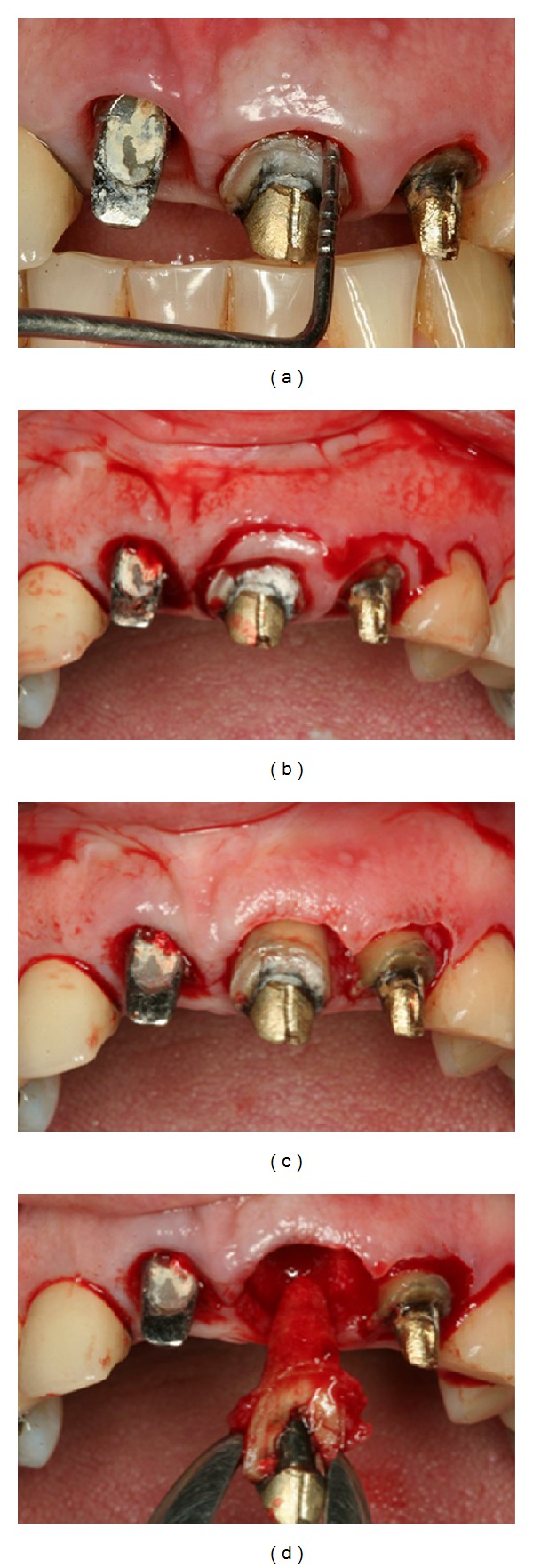
(a, b, c) Clinical crown lengthening of the anterior teeth to correct the uneven gingival level. (d) Atraumatic extraction of the left central incisor.

**Figure 6:**
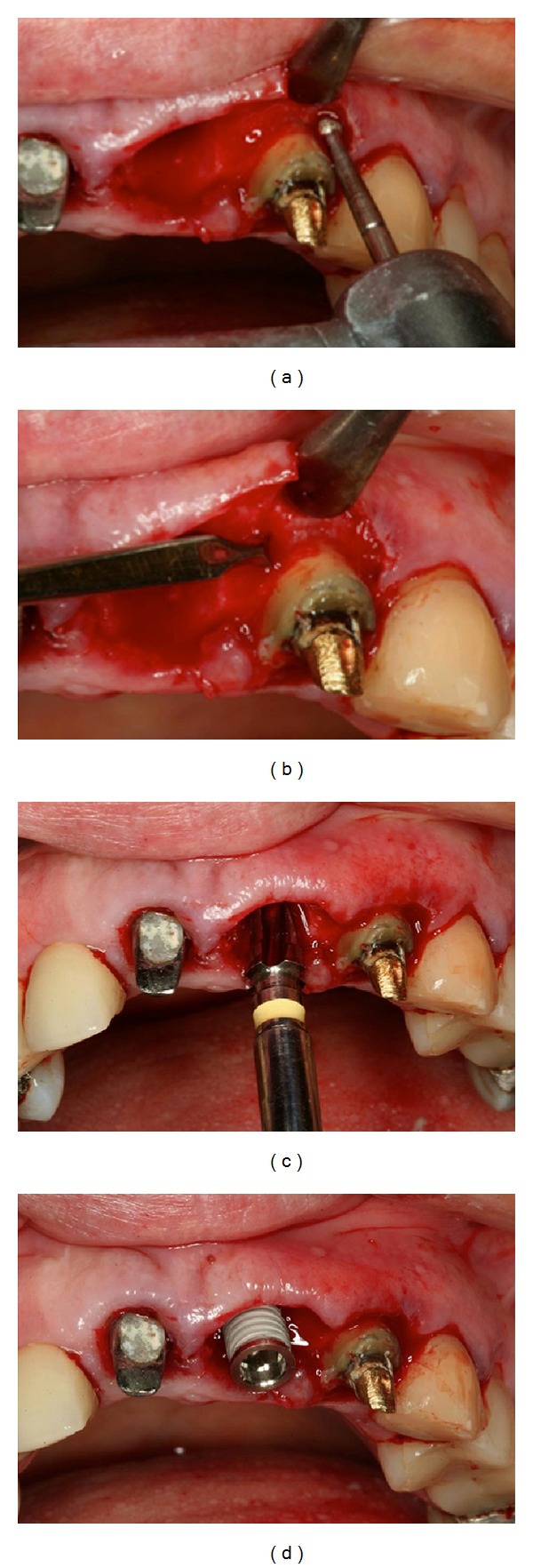
(a, b) After tooth extraction, root planing and curettage of the alveolus and bone removal for crown lengthening, with the aid of a chisel, were performed. (c, d) A 4.3 mm internal hexagon implant was placed 3 mm below the alveolar bone crest in the direction of palatal cortical bone.

**Figure 7:**
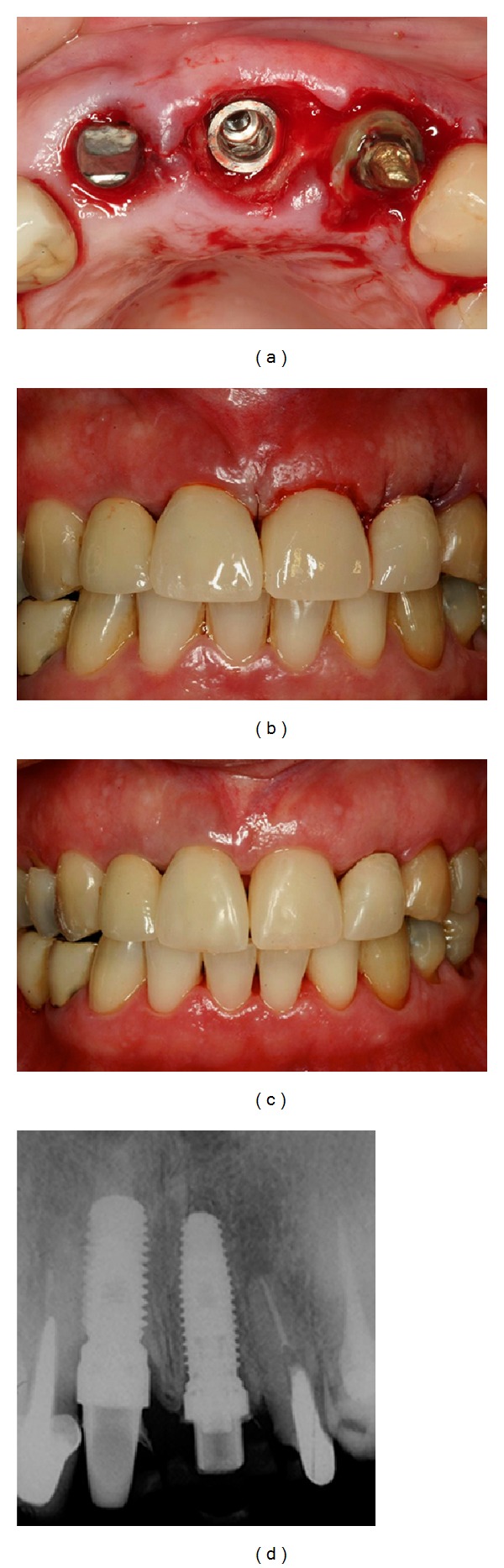
(a) Occlusal view of the implant placed. (b, c) Immediate postoperative views with provisional resin crown in the central and lateral incisor teeth. (d) Periapical radiography after implant installation.

**Figure 8:**
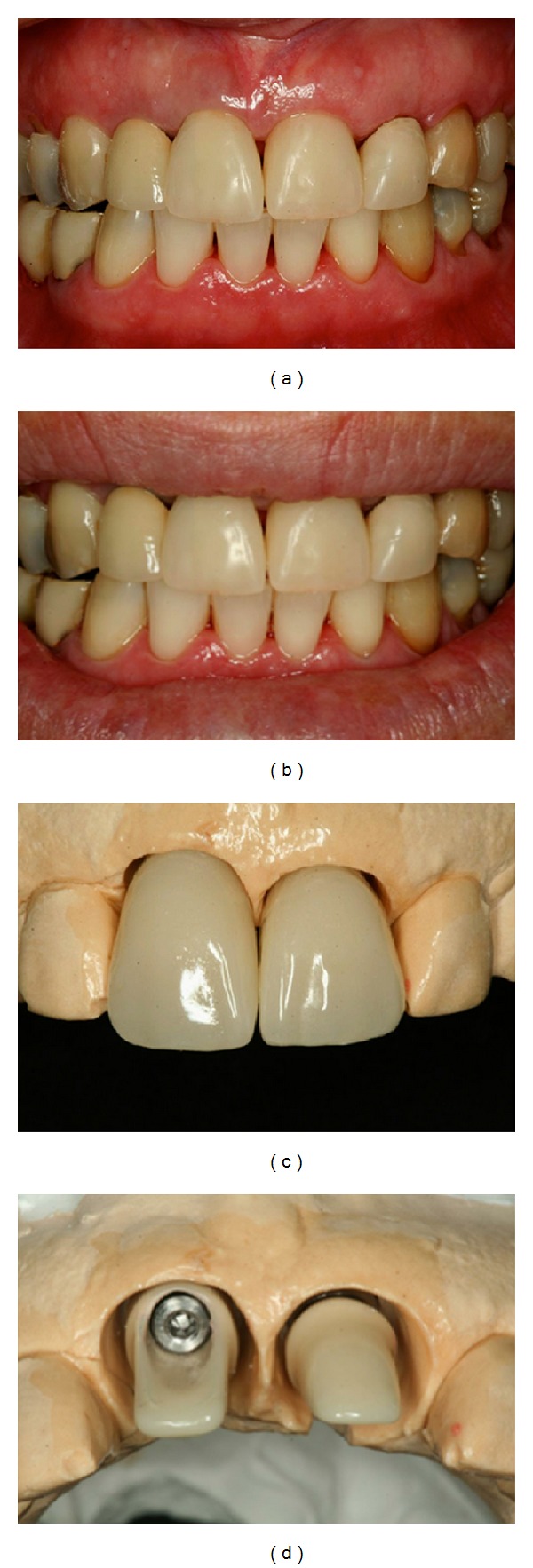
(a, b) 4 months after the implant placement in the left central incisor. (c, d) Application of ceramic Ivoclar press on the abutments to improve the aesthetic results.

**Figure 9:**
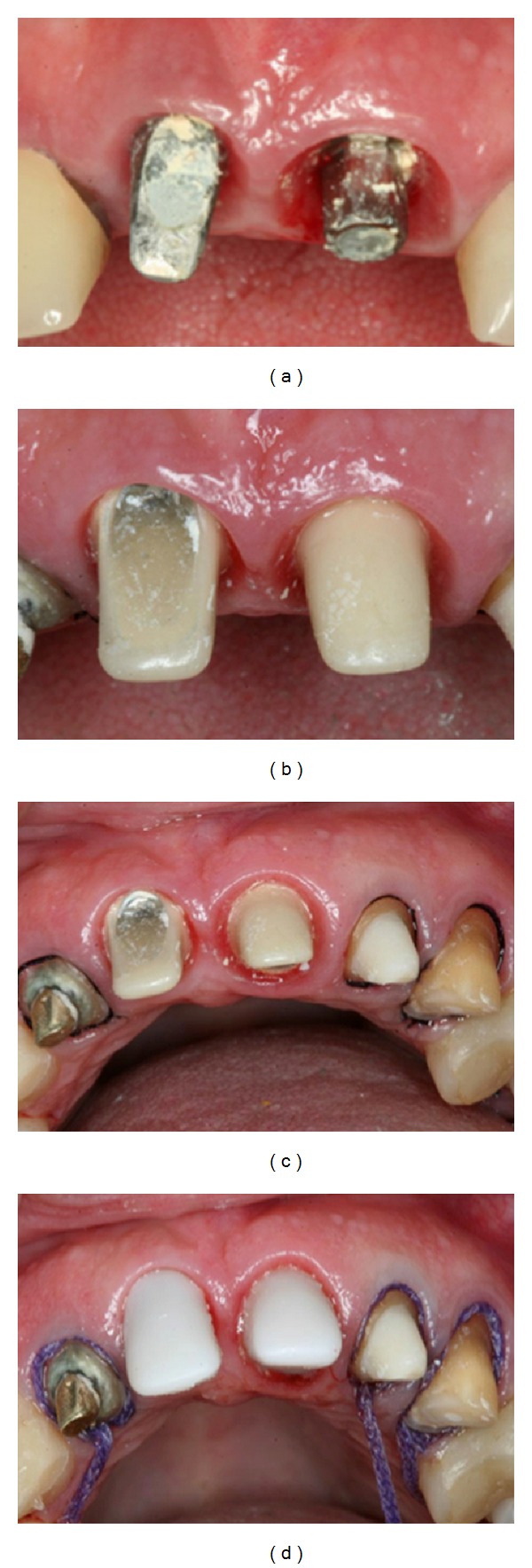
IPS and max press copings used to restore the maxillary central and left incisors and left canine.

**Figure 10:**
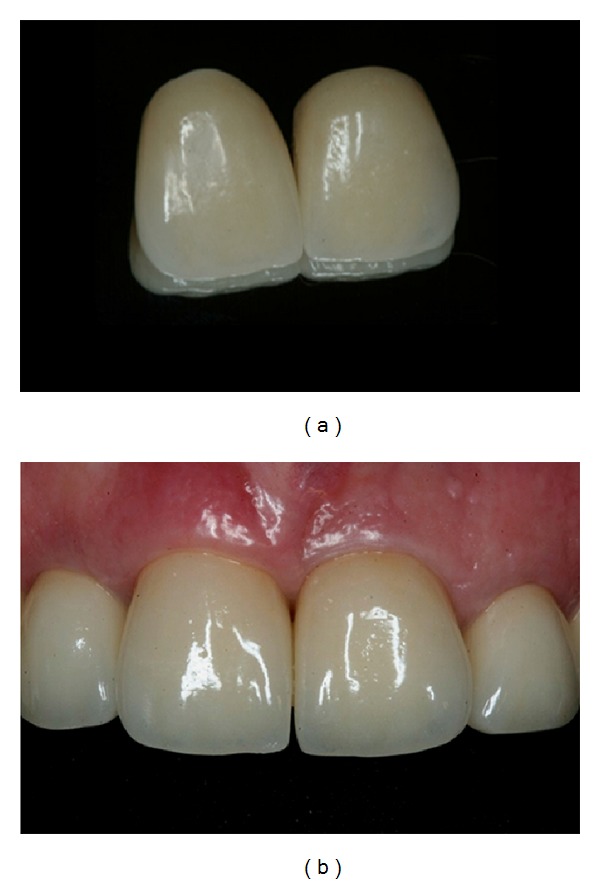
Buccal view of the implant crown and its excellent aesthetic results at a 12-month follow-up.

**Figure 11:**
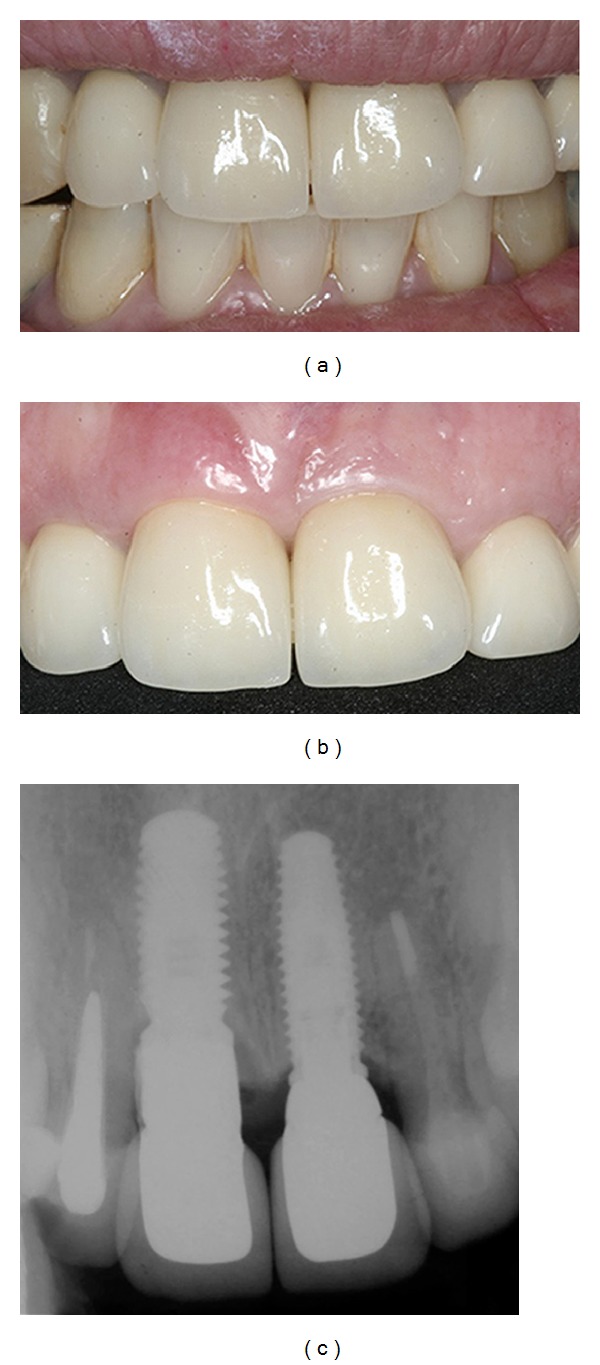
(a, b) Three years follow-up; the result showed esthetic excellence of the definitive prosthesis; (c) periapical radiograph. Three-year follow-up showing the accurate fit between the abutments and implants and between the crowns and abutments.

**Figure 12:**
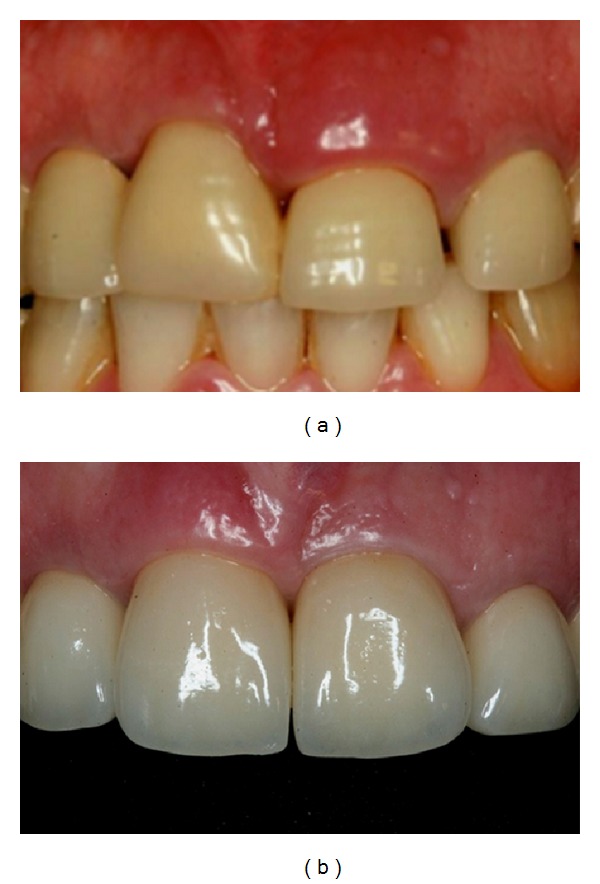
(a) Initial and (b) final clinical situations.
